# Boarding and Day School Students: A Large-Scale Multilevel Investigation of Academic Outcomes Among Students and Classrooms

**DOI:** 10.3389/fpsyg.2020.608949

**Published:** 2021-01-05

**Authors:** Andrew J. Martin, Emma C. Burns, Roger Kennett, Joel Pearson, Vera Munro-Smith

**Affiliations:** ^1^School of Education/School of Psychology, University of New South Wales, Sydney, NSW, Australia; ^2^Department of Educational Studies, Macquarie University, Macquarie Park, NSW, Australia; ^3^The Future Project, The King’s School, Sydney, NSW, Australia

**Keywords:** boarding, residential, motivation, engagement, achievement, science

## Abstract

Boarding school is a major educational option for many students (e.g., students living in remote areas, or whose parents are working interstate or overseas, etc.). This study explored the motivation, engagement, and achievement of boarding and day students who are educated in the same classrooms and receive the same syllabus and instruction from the same teachers (thus a powerful research design to enable unique comparisons). Among 2,803 students (boarding *n* = 481; day *n* = 2,322) from 6 Australian high schools and controlling for background attributes and personality, we found predominant parity between boarding and day students in their motivation, engagement, and achievement. We also found that classroom-average motivation, engagement, and achievement was not significantly affected by the number of boarders (relative to day students) in the classroom. In addition, the effects of boarding were generally not moderated by students’ background or personality attributes. We conclude that boarders have academic opportunities and outcomes that are comparable to their day student counterparts. Implications for students, teachers, and parents are discussed.

## Introduction

Boarding schools^1^ constitute a major mode of education in many countries. For example, in Australia (the site of the present study) there are an estimated 170 schools with boarding students, and 470 schools in the United Kingdom and 340 schools in North America that accommodate boarding students (Martin et al., 2014). There has been a growing body of research into boarding school, particularly in Australia (the site of the present study). This research has been quantitative and qualitative and contributed to increasing understanding of boarders, their academic and social-emotional wellbeing outcomes, and the factors contributing to these. Research in this area is important because boarding (and other residential education settings) is often a necessary educational pathway for many students for a variety of reasons (e.g., living in remote areas, parents working overseas, choosing education outside home country, etc.). Indeed, investigating boarding school effects involves quite a unique research design in that boarding and day students are educated in the same classrooms, taught by the same teachers, and receive the same instruction and syllabus. Thus, boarders may be considered something of a “treatment” group and day students something of a “comparison” group, with most curricular classroom and instructional features held constant.

In numerous ways, the present study adds to research findings about boarding school students. First, it explores in a large-scale sample, the role of boarding in students’ domain-general academic motivation and engagement (i.e., motivation and engagement in school generally). Second, it extends the domain-general motivation and engagement research by also investigating the role of boarding in students’ domain-specific (science) motivation, engagement, and achievement. Third, it augments prior multilevel research (that focused on students nested within boarding houses and schools; Martin et al., 2016) by conducting multilevel research investigating student- and classroom-level effects of boarding status on academic outcomes—e.g., whether the number of boarders relative to the number of day students in a class affects classroom-average motivation, engagement, and achievement.

**FIGURE 1:**
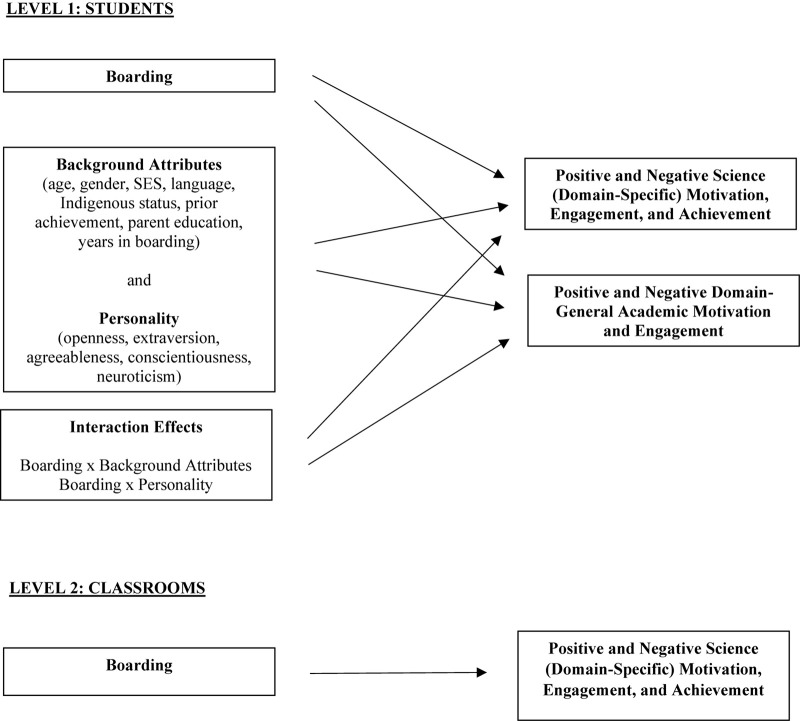
Multilevel path model to be tested.

Figure 1 presents the multilevel model we apply to address these three issues. At Level 1 of this figure are the student-level associations to be tested. Here boarding status (no/yes; or, day/boarding) predicts science motivation, engagement, and achievement and also predicts domain-general motivation and engagement. Importantly, boarding status is a predictor of these outcomes alongside students’ background attributes (e.g., age, gender, Indigenous status, etc.) and their personality in order to ascertain the role of boarding beyond the role of background attributes and personality. At Level 1 also, interaction effects are tested that explore whether boarding status effects vary as a function of background attributes and personality (e.g., whether boarding status effects vary as a function of different age groups). Level 2 explores boarding effects on science motivation, engagement, and achievement at the classroom level—that is, whether the proportion of boarders in a science classroom predicts class-average science outcomes. Importantly, multilevel modeling disentangles Level 2 from Level 1 effects; thus, Level 2 findings shed light on class-average effects beyond individual student effects.

## Theoretical Perspectives

There are numerous theoretical frameworks that can inform thinking about the effects of boarding. Relevant to this study’s substantive foci are ecological systems, human capital, critical race, social identity, and extracurricular activity theories.

### Ecological Systems, Human Capital, and Critical Race Frameworks

Ecological systems theory emphasizes the ongoing person-environment interactions that shape human development (Bronfenbrenner, 2001, 2005). Under this theory:

… human development takes place through processes of progressively more complex reciprocal interaction between an active, evolving biopsychological human organism and the persons, objects, and symbols in its immediate external environment. To be effective, the interaction must occur on a fairly regular basis over extended periods of time (Bronfenbrenner and Morris, 1998, p. 996).

We contend that boarding represents a somewhat intensive and on-going process of interactions between student and environment—interactions that have potential to shape students’ academic outcomes (Martin et al., 2014, 2016). In fact, given the salient contextual aspects of boarding, it is perhaps not surprising that Bronfenbrenner (1970) conducted one of the earliest formal investigations of boarding effects. According to Bronfenbrenner, because the boarding context plays a different role in shaping children’s academic development, it is conceivable boarding students’ academic outcomes may differ from those of day students.

Bass (2013) draws on Bourdieu’s (1986) ideas around social and educational capital to explore the potential that boarding may (or may not) hold for improving life chances for disadvantaged youth through opportunities for meeting their social and educational needs. At the same time, however, these capital theories and their positive contentions do not always apply to some groups of boarding students, e.g., due to a lack of supporting data (Guenther and Fogarty, 2018). Also, human capital theory has been connected with other pertinent theories such as critical race theory (Aleman, 2013) that might suggest potentially challenging perspectives on boarding effects, particularly for some student groups. For example, critical race theory has been applied as a lens to understand boarding school for Indigenous students (Benveniste et al., 2019). For these students, boarding school may reproduce dominant cultural values through their daily practices, policies and procedures that are not appropriately sensitive or supportive of Indigenous students and their cultural and social-emotional needs.

### Social Identity Theory

Social identity theory is also relevant. Individuals’ self-concepts are based on their membership to their social group (Tajfel, 1978; Tajfel and Turner, 1986). Social identities are most influential when the individual has strong emotional connections to a group and when membership in a particular group is considered by the individual to be central to their self-concept. The individual garners self-esteem through affiliation with the group, typically through influential processes such as within-group assimilation (pressure to conform to the group’s norms) and intergroup bias (favorably appraising one’s own group relative to other groups). These processes are particularly powerful in peer groups (Leaper, 2011). This being the case, there have been applications of social identity theory to the educational context. Mavor et al. (2017), for example, have described how the “self” is not a fixed entity among students, but amenable to variation as a function of change in experience, including formal and informal learning at school. These ideas are particularly relevant when considering students who experience a boarding context for their residential experience and who are taught within specific classrooms for their educational experience. The present study and its multilevel design are ideally placed to investigate these processes in terms of the development of boarding and day students’ academic outcomes at school generally and also in science classrooms.

### Extracurricular Perspectives

Extracurricular activity is any out-of-class involvement that absorbs students’ energy, time, and attention (Marsh and Kleitman, 2002), and as such, boarding can be considered a form of extra-curricular activity. The “identification/commitment” model of extracurricular activity (Marsh, 1992) proposes that school-based extracurricular activities can “improve school identification, involvement, and commitment in a way that enhances narrowly defined academic outcomes” (Marsh and Kleitman, 2002, p. 471). It has been found that school-based extracurricular activities are more likely to increase students’ affiliation with the school (Fredricks and Eccles, 2005). Following from this, Martin et al. (2014) proposed that context-specific affiliation (e.g., school affiliation) boosts students’ identification with and commitment to that context, resulting in positive academic outcomes. They further proposed that boarding may afford greater student activity at and with the school—indeed, being resident at school may also involve a greater requirement or expectation to be involved in extra-curricular activities. Thus, it is possible that one’s boarding status is linked with adaptive academic outcomes, consistent with what might be hypothesized for school-based extracurricular activity under the “identification/commitment” perspective (Marsh and Kleitman, 2002). However, the counterpoint to this is that time spent in one activity comes at the expense of potential development in other parts of life (Marsh and Kleitman, 2002); for example, boarding may deprive students of necessary development opportunities (such as what they might gain at home), and potentially have negative effects.

## Research Relevant to the Boarding Experience

To date, research into boarding has revealed a somewhat mixed body of results, finding positive, negative, or generally null (or equivocal) effects in boarders’ academic and social and emotional outcomes. It is also the case there are different student groups for whom boarding is a more salient educational option and research has identified effects particular to these students as well. This research is briefly reviewed.

### Positive and Negative Effects of Boarding

The Association of Boarding Schools (The Association of Boarding Schools (TABS), 2003, 2013) compared the experiences of U.S boarding students and day students. Findings showed that boarding students were more likely to report they were satisfied with their academic experience and were more likely to report that school prepared them for college. In a qualitative investigation, White’s (2004) study of Anglo-Australian and overseas students suggested that boarding instills independence and acceptance of cultural diversity. Also, in qualitative work, Bass (2013) found that boarding for disadvantaged students enhanced their exposure to social and educational capital. An Australian study by Martin et al. (2014) found predominant parity between boarding and day students (described below), but where small effects were identified, they favored boarders. These studies thus suggest potentially positive outcomes for boarding students.

There is also research documenting negative effects of boarding for some children. Lester and Mander (2020) investigated mental health and wellbeing among high school boarders (boys) as they transitioned to and into boarding school. They found increases in emotional problems among boarding students over time. They also found that academic motivation declined over time; however, this was the case for both boarders and day students. In longitudinal research, Mander and Lester (2017) found that boarding and day students reported increases in depression, anxiety, and emotional symptoms between Grades 7 and 9, but that boarding students reported higher levels of anxiety and stress than day students at the end of Grade 8. It has also been documented how some boarding schools are contexts perpetuating institutional and societal power structures and problematic ideologies—such as those around gender (Khan, 2010; Finn, 2012; also see Duffell, 2000; Schaverien, 2011 for other analyses of negative boarding effects).

### Null Boarding Effects

There is also research showing there is not a major difference in educational outcomes when comparing boarding and day school students. As noted above, Martin et al. (2014) conducted a large-scale Australian study and found relatively few differences (with small effect sizes) in academic wellbeing (e.g., domain-general academic motivation and engagement) and personal wellbeing (e.g., peer relations, mental health, etc.) when comparing boarding and day students in the same school. In a similar vein, in a longitudinal study of students transitioning from day to boarding status, Downs (2002) found no major changes in self-concept through this transition. Behaghel et al. (2017) found that disadvantaged students in boarding initially experienced low levels of wellbeing, but their wellbeing adjusted during their boarding experience. They also found boarders experienced academic gains 2 years after commencing boarding, but this effect did not generalize across students (it was stronger for students higher in initial academic ability).

### Insights From Particular Student Groups

It is also the case that particular student groups have a more long-established history of attending boarding school. On the international stage, overseas students are one such group (usually because their parents are working in another country). In the Australian context (the site of the present study), boarding has been a major educational pathway for Indigenous students, with most research identifying mixed yields in the boarding school experience for these students. For example, in a study of Indigenous girls in a residential college it was found they enjoyed their residential experience and the new friendships developed, but also found that homesickness and lifestyle restrictions were a challenge for the girls (English and Guerin, 2017). These findings were similar to a study by MacDonald et al.(2018; see also Guerin and Pertl, 2017) where school leaders and Indigenous students reported that boarding allowed enhanced career opportunities and health outcomes, but that there were issues to navigate to attain these outcomes such as homesickness, racism, and post-school transition difficulties. Guenther and Fogarty(2018; see also Guenther et al., 2020) identified the positive possibilities of boarding school for Indigenous students, but also noted the evidence does not always support the positive potential. They suggested that when interpreting Indigenous students’ development through cultural and human capital lenses, there emerge potential problems and difficulties in boarding for Indigenous students that have significant implications for educational policy. Indeed, quantitative research among high school Indigenous boarders supports this, finding lower scores on resilience and psycho-social wellbeing. Also, when these students transitioned back to their community, they reported less connectedness with family and community and even lower levels of resilience and psycho-social wellbeing (Redman-MacLaren et al., 2019).

### Summary and Focus of This Study

Taken together, it is evident the diversity of research methodologies that have examined the experiences and outcomes of boarding, has yielded varied findings. Each has informed a distinct aspect of the boarding phenomenon, both positive and negative. The present study adds to what is known by addressing two novel dimensions in this space. First, given that boarding students are typically taught in the same classes as day students, what is the effect of the relative proportion of boarders in a class on class-average academic outcomes? For example, does the presence of relatively more (or fewer) boarders in a class affect class-average outcomes? Second, prior research has investigated the effects of boarding on domain-general academic outcomes (e.g., motivation in school generally), but we do not know if such findings generalize to specific school subjects. We therefore investigate the effects of boarding on domain-general (in school, generally) and domain-specific (in science) academic outcomes. Figure 1 shows the multilevel processes we investigate (described above).

## Domain-General and Domain-Specific Outcomes, Background Attributes, and Multilevel Considerations

### Target Domains and Outcomes Under Focus

As noted, we focus on domain-general (i.e., in school, generally) academic outcomes and domain-specific academic outcomes. Our domain-specific focus is science—specifically motivation, engagement, and achievement in science. Exploring these issues in science is somewhat topical because there are concerning trends in science achievement and science pathways (especially among “Western” nations). In Australia, for example, achievement in science declined in the 2015 Trends in International Mathematics and Science Study (TIMSS; Thomson et al., 2016). In the Programme for International Student Assessment (PISA), the long-term change in Australia’s mean performance in science over the period of its participation demonstrates one of the largest decreases among PISA-participating countries (OECD, 2020). Also, science participation and enrollments among senior school students demonstrate long-term decline (Office of the Chief Scientist, 2014) and there is concern about students’ declining interest in science in high school (Tröbst et al., 2016). Thus, motivation, engagement, and achievement have been identified as outcome targets for improvement in science and there have been recommendations for researchers to explore factors that may be implicated in these outcomes (Ross and Poronnik, 2013; Abraham and Barker, 2015). Our study therefore investigates the role of educational context (specifically, boarding vs. day status) as one potential factor. Importantly, to ascertain if potential boarding status effects are distinct to science or not, we also assess the role of boarding status in motivation and engagement for school in general. In operationalizing motivation and engagement as “outcomes” in this study, we do recognize that they can also be considered as “input” or predictor factors for achievement and other academic outcomes. We herein position them as outcomes because it is more feasible that boarding status and background attributes such as gender, SES, etc. predict motivation and engagement, than vice versa. Thus, motivation and engagement can be either a means to desirable outcomes, *or* desirable outcomes in their own right—and it is the conceptualizing, research questions, and research design that determine where in the educational process they are modeled (Marsh and Martin, 2011; Martin, 2012)—viz. “outcomes” in the case of the present study.

Because we seek to systematically build on the recent large-scale quantitative study by Martin et al. (2014), we adopt the main motivation and engagement measures employed by them; namely, positive motivation (e.g., self-efficacy), positive engagement (e.g., persistence), negative motivation (e.g., anxiety), and negative engagement (e.g., self-handicapping). These are all operationalized through the Motivation and Engagement Scale that has domain-general (Martin, 2007) and domain-specific (including in science; Green et al., 2007) forms. For achievement, we administer an in-survey science test that assesses students on the extent to which they have acquired core information from the state science syllabus.

### Background Attributes Important to Consider

It is possible that boarding status may be associated with various student background attributes that are also linked with motivation, engagement, and achievement. To understand the unique effects of boarding, it is thus important to include such attributes in modeling in order to partial out their potential influence. Martin et al. (2014) identified numerous such factors, including age, gender, socio-economic status, language background, Indigenous status, parent education, prior achievement, and personality. For example, they found that alongside boarding status, parents’ education, prior achievement, conscientiousness, agreeableness, and openness all positively predicted positive motivation—while prior achievement, conscientiousness, and agreeableness negatively predicted negative motivation (and neuroticism positively predicted negative motivation). Furthermore, if boarding represents a distinct educational ecology and socializing environment (Bronfenbrenner, 1970; Holden et al., 2010), then time spent in that environment (i.e., years in boarding school) may affect one’s identification with and internalization of that environment, including academic effects of the experience. Thus, background attributes do explain variance in this study’s academic outcomes beyond the effects of boarding status. Accordingly, alongside the predictive role of boarding status, these background attributes are also included in the present study as predictors of motivation, engagement, and achievement (i.e., shared variance is controlled for; see Figure 1).

Furthermore, according to Martin et al. (2014), it is also possible that background attributes may moderate the effects of boarding. For instance, perhaps boarding effects vary as a function of students’ age, Indigenous status, personality, etc. In Australia, boarding is identified as one means by which distant students (e.g., Indigenous, rural, or remote) can access education (e.g., Curto and Fryer, 2011; MacDonald et al., 2018; Osborne et al., 2019; Guenther et al., 2020). Also, we earlier identified research revealing a negative history of boarding school for some students and in part this has been attributed to the commencement of boarding at a young age (Duffell, 2000). Although our study is conducted in high schools, we can test if age moderates the effects of boarding on academic outcomes. Or, it may be that the somewhat social nature of residential education is better suited to students high in extraversion. Thus, we include interaction terms (e.g., boarding status × Indigenous status, boarding status × age, boarding status × extraversion, etc.) to test for the potential moderating role of the study’s background attributes (see Figure 1).

### Multilevel Considerations

There is widespread recognition of how important it is to analyze hierarchical data in appropriate ways (Marsh et al., 2012). In our study we have students nested within classrooms and therefore conduct multilevel modeling to account for this and to understand variance attributable to student- and classroom-levels. There are known statistical biases associated with single-level research designs (e.g., dependencies within groups; confounding of within- and between-group variables) and multilevel approaches aim to resolve these biases (for discussions see Goldstein, 2003; Marsh et al., 2008; Raudenbush and Bryk, 2002). To our knowledge only one study has investigated boarding from a multilevel perspective—Martin et al. (2016) investigated motivation and social climates among students nested within boarding houses that were nested within schools (thus, student-, house-, and school-level effects).

Our study differs from the Martin et al. (2016) work by exploring student- and classroom-level effects. Specifically, we investigate whether the proportion of boarding students (relative to day students) in a class has a significant bearing on class-average motivation, engagement, and achievement. Multilevel modelers have established the reciprocity of individual and group dynamics: individuals can affect the group to which they belong and in turn the group can affect these individuals (Raudenbush and Bryk, 2002; Goldstein, 2003; Marsh et al., 2008). This raises the question as to whether a critical mass of boarding students in a classroom affects overall class-average outcomes. For example, does the distinct socialization experience of boarding (Holden et al., 2010) lead to a cohesion or collective identity among boarders in a classroom such that they evince distinct effects relative to day student counterparts in the same classroom? By capturing data on science motivation, engagement, and achievement in science classrooms, our research design could address this question.

When conducting multilevel modeling it is also important to establish whether climate or context effects are being investigated. Climate refers to shared perception of a characteristic of the group (e.g., classroom) that is common to members (e.g., students) in that group. For climate variables, the group referent is usually explicit in the item, indicator, or question (e.g., “… students in this classroom try hard”; Marsh et al., 2012). However, when the item referent is the individual (e.g., “I try hard”) and the item is aggregated “up” to also create a classroom-level variable, it is known as a context effect (Marsh et al., 2012). As is evident in Materials below, in our study all variables at student- and classroom-level are context factors.

## Aims of the Present Study

There were three main aims of the present investigation. The first aim was to explore, in a large-scale sample, the role of boarding in students’ domain-general academic motivation and engagement. The second aim was to also explore the role of boarding in students’ domain-specific (science) motivation, engagement, and achievement. The third aim was to investigate the association between the proportion of boarders in a classroom (relative to day students) and classroom-average motivation, engagement, and achievement—beyond the student-level motivation, engagement, and achievement relevant to the first two aims. Figure 1 presents the multilevel path model addressing these three aims.

## Methods

### Sample

Participants were 2,803 high school students from 6 Australian schools that comprised both boarding and day students. Students were surveyed in 224 science classrooms (mean class size = 11.68 students; sufficient to estimate classroom effects and not unduly disproportionate to the staff-to-student ratio for high schools in the independent school sector, taking into account non-teaching staff numbers, non-participation, student absences, and any students not receiving parental participation consent; Australian Bureau of Statistics, 2019). Seventeen percent (*n* = 481) of students were boarders; 83% (*n* = 2,322) were day students. Thirty-five percent of boarders had been boarding for less than 1 year, 31% for 1–2 years, and 34% for 3 years and over.

All schools were independent schools (i.e., not government or systemic) and located in Sydney, New South Wales (Australia’s most populous state). The average school size was 1,801 total students enrolled. Regarding the socio-demographics of the school, in 2018 (the year data were collected), 23% of the students enrolled within the 6 schools spoke a language other than English at home and 1% of students enrolled within the 6 schools identified as Aboriginal/Torres Strait Islander (Australian Curriculum Assessment Reporting Authority (ACARA), 2020a). For school socio-economic status, the average Index of Community Socio-educational Advantage (ICSEA) score for the schools sampled is 1,145 (compared to the national *M* = 1,000; Australian Curriculum Assessment Reporting Authority (ACARA), 2020a). Regarding numeracy achievement in the National Assessment Program—Literacy and Numeracy (NAPLAN), the mean numeracy score of the 6 schools sampled was *M* = 626 (compared to the national *M* = 572; Australian Curriculum Assessment Reporting Authority (ACARA), 2020a,b). Regarding literacy in NAPLAN, the mean literacy score of the 6 schools sampled was *M* = 593 (compared to the national *M* = 553; Australian Curriculum Assessment Reporting Authority (ACARA), 2020a,b). Taken together, these trends indicate that the 6 schools perform above the national average.

Of the 6 schools, 1 school was co-educational, 1 school was a single-sex girls’ school, and 4 schools were single-sex boys’ schools. This being the case, the majority of students were boys (92%). This is disproportionate, but we point out that: (a) multigroup (male vs. female) confirmatory factor analysis of the motivation and engagement measures suggested scalar invariance (the minimum criterion for invariance; Van de Schoot et al., 2012) as a function of gender, with no change greater than 0.01 for CFI or greater than 0.015 for RMSEA (Chen, 2007; Cheung and Rensvold, 2002), (b) in preliminary analyses (see Table 2) there were no gender differences in the proportion of boarders to day students, (c) there were no correlations (Table 2), predictive main effects (Table 3B), or moderating effects (viz. boarding/day status × gender) between gender and outcome variables that attained our minimum benchmark for interpretability, (d) as we show below our findings align with those of Martin et al. (2014) whose research design we followed and which comprised relatively equal numbers of boys and girls, and (e) we selected a random sample of 8% boys to match the 8% girls and re-ran the final Step 3 model (see section “Data Analysis,” below), also finding that the only three boarding effects approaching our minimum benchmark for interpretability were the same three boarding effects that approached or attained our minimum benchmark for interpretability in the full sample (Table 3B). We thus tentatively conclude that our gender composition did not unreasonably impact factors and empirical associations in this study.

The average age of students was 14.14 years (*SD* = 1.29; boarding students *M* = 14.47, *SD* = 1.25; day students *M* = 14.07, *SD* = 1.29). Eleven percent of the sample were from a non-English speaking background (boarding students 10%; day students 11%). Six percent were Indigenous students (boarding students 9%; day students 5%). Students rated their mother’s and father’s level of education from 1 (“no formal qualifications”) to 6 (“university undergraduate or higher degree”) (sample *M* = 5.14, *SD* = 1.28; boarding students *M* = 4.71, *SD* = 1.45; day students *M* = 5.23, *SD* = 1.22). Students’ socio-economic status (SES) based on the Australian Bureau of Statistics Index of Relative Socio-Economic Advantage and Disadvantage classification (sample *M* = 1120, *SD* = 65; boarding students *M* = 1035, *SD* = 93; day students *M* = 1137, *SD* = 41) was higher than the national average (*M* = 1000, *SD* = 100). As shown in Figure 1, each of these background factors was included in formal modeling to control for their influence in effects.

### Procedure

Human ethics approval was provided by the lead researcher’s institution. Approval was then received from each school principal agreeing to their school’s participation. Parents/carers and participating students then both provided consent. The online survey of motivation and engagement (as well as a science test) was administered to students during a science lesson in the second term (of four school terms) of 2018. Students were instructed to respond to the survey and test on their own. They were also informed that teachers could provide assistance with any procedural aspects of the process, but that teachers could not help students in answering specific items.

### Materials

#### Science Motivation and Engagement

Science motivation and engagement were assessed using the Motivation and Engagement Scale—High School (MES-HS; Martin, 2015), adapted to science (Green et al., 2007). Positive motivation in science comprised mastery orientation (e.g., “I feel very pleased with myself when I do well in this science class by working hard”; 4 items), self-efficacy (e.g., “If I try hard, I believe I can do well in this science class”; 4 items), and valuing (e.g., “Learning in this science class is important”; 4 items). Positive science engagement comprised task management (e.g., “When I study for this science class, I usually try to find a place where I can study well”; 4 items), planning behavior (e.g., “I try to plan things out before I start working on homework or assignments for this science class”; 4 items), and persistence (e.g., “If I don’t give up, I believe I can do difficult schoolwork in this science class”; 4 items). Negative science motivation was measured with anxiety (e.g., “When exams and assignments are coming up in this science class, I worry a lot”; 4 items), failure avoidance (e.g., “Often the main reason I work in this science class is because I don’t want to disappoint my parents”; 4 items), and uncertain control (e.g., “I’m often unsure how I can avoid doing poorly in this science class”; 4 items). Negative science engagement was assessed via disengagement (e.g., “I’ve pretty much given up being involved in things in this science class”; 4 items), and self-handicapping (e.g., “I sometimes put assignments and study off until the last moment, so I have an excuse if I don’t do so well in this science class”; 4 items). Students rated items on a scale of 1 (Strongly Disagree) to 7 (Strongly Agree). In previous research, these measures are shown to be normally distributed, reliable, and validated with educational outcomes (for review see Liem and Martin, 2012), including in science (Green et al., 2007). Because the science motivation and engagement items in this study were directly relevant to the classrooms in which students were responding to the survey (i.e., their science lesson/class), we modeled the science motivation and engagement factors at Level 1 (L1, student-level) and at Level 2 (L2, class-level).

For this study we focused on the 4 higher order MES factors (positive motivation, negative motivation, positive engagement, negative engagement) that were estimated by (a) aggregating (mean scoring) the items of each first order MES factor (e.g., the 4 items for self-efficacy) to create 11 MES scale scores (e.g., a self-efficacy scale score) and (b) using these scale scores to create an error-adjusted mean score for each of the 4 higher order factors. Error adjusted scores were derived using the following formula: σ_h_^2^^∗^ (1 −ω_h_), where σ_h_^2^ is the estimated variance of and ω_h_ is the reliability estimate of the motivation and engagement factor (h) at either L1 (student) or L2 (class; Hayduk, 1987; see also Cole and Preacher, 2014). Error-adjusted scores were used because they help avoid unreliable standard errors and reduce the risk of inflated parameter estimates (Cole and Preacher, 2014). This yielded standardized loadings as follows: positive science motivation, L1 = 0.96 and L2 = 0.98; positive science engagement, L1 = 0.94 and L2 = 0.95; negative science motivation, L1 = 0.93 and L2 = 0.87; and negative science engagement, L1 = 0.92 and L2 = 0.93. As shown in Table 1, these factors were normally distributed. Table 1 also shows acceptable reliability (McNeish, 2018) at L1 and L2 for positive motivation (L1ω_h_ = 0.83; L2ω_h_ = 0.98), positive engagement (L1ω_h_ = 0.84; L2ω_h_ = 0.96), negative motivation (L1ω_h_ = 0.69; L2ω_h_ = 0.87), and negative engagement (L1ω_h_ = 0.72; L2ω_h_ = 0.95).

**TABLE 1:** Descriptive and measurement properties for outcome variables.

	Mean	*SD*	Skew	Kurtosis	ICC	Level 1 (student) omega	Level 2 (class) omega
**Domain general**							
- Positive motivation	5.72	1.01	−1.09	1.81	–	0.76	–
- Negative motivation	3.94	1.26	0.08	−0.02	–	0.61	–
- Positive engagement	5.18	1.14	−0.53	0.17	–	0.83	–
- Negative engagement	3.79	1.38	−0.07	−0.51	–	0.57	–
**Science**							
- Positive motivation	5.61	0.96	−1.03	1.24	0.14	0.83	0.98
- Negative motivation	3.47	1.04	0.18	−0.24	0.08	0.69	0.87
- Positive engagement	4.89	0.97	−0.45	0.21	0.09	0.84	0.96
- Negative engagement	2.48	1.11	0.79	0.20	0.14	0.72	0.95
- Achievement	0.00	1.00	−0.14	−0.60	0.31	0.69	0.98

*Domain-general academic motivation and engagement factors are only modeled at L1 (student-level; hence no L2 or ICC statistics available); achievement is a single formative standardized score (see section “Materials”). SD, standard deviation; ICC, intraclass correlation.*

**TABLE 2:** Multilevel correlations: students and classrooms.

		Domain-general (DG) academic motivation and engagement				Domain-specific science (Sc) motivation and engagement				
			
	Boarding (N/Y)	Positive motivation	Negative motivation	Positive engagement	Negative engagement	Positive motivation	Negative motivation	Positive engagement	Negative engagement	Achievement
**Level 1 (student)**										
Age	0.14***	−0.12**	0.04	−0.15***	0.19***	−0.18***	0.04	−0.18***	0.20***	−0.04
Gender (M/FM)	−0.08	0.01	0.04	−0.05*	0.08*	0.01	0.08*	0.01	0.04	−0.04
Socio-economic status	−0.59***	0.05*	−0.06	0.01	−0.03	0.04	−0.04	0.01	−0.04	0.10**
Non-English speaking	0.01	0.01	0.07	0.04	−0.02	0.04	0.02	0.04	−0.01	0.07*
Indigenous	0.07	−0.01	0.02	−0.01	0.03	0.01	−0.01	0.01	−0.01	−0.03*
Parent education	−0.12***	0.07	−0.05	0.04	-0.04	0.09***	−0.04	0.05	−0.07	0.16***
Prior achievement	−0.14***	0.24***	−0.21	0.23***	−0.18***	0.27***	−0.28***	0.22***	−0.28***	0.42***
Neuroticism	−0.01	−0.14***	0.52	−0.06***	0.36***	−0.10***	0.43***	−0.10***	0.19***	−0.09**
Openness	−0.02	0.52***	−0.12	0.45***	−0.16***	0.43***	−0.19***	0.41***	−0.35***	0.15***
Conscientiousness	−0.02	0.46***	−0.10	0.51***	−0.25***	0.32***	−0.19***	0.41***	−0.35***	0.16***
Agreeableness	−0.05***	0.40***	−0.05	0.36***	−0.08***	0.27***	−0.10***	0.30***	−0.24***	0.08**
Extraversion	0.01	0.36***	−0.08	0.34***	−0.16***	0.24***	−0.11**	0.30***	−0.21***	0.01
DG positive motivation	−0.02		−0.10	0.74***	−0.29***	0.57***	−0.26***	0.52***	−0.46***	0.22***
DG negative motivation	0.02			0.03	0.50***	−0.12***	0.63***	−0.08***	0.25***	−0.18***
DG positive engagement	0.02				−0.40***	0.47***	−0.18***	0.73***	−0.45***	0.12***
DG negative engagement	−0.01					−0.28***	0.43***	−0.44***	0.47***	−0.11***
Sc positive motivation	−0.05						−0.24***	0.73***	−0.74***	0.33***
Sc negative motivation	0.01							−0.21***	0.45***	−0.24***
Sc positive engagement	−0.01								−0.65***	0.18***
Sc negative engagement	0.07*									−0.34***
Sc achievement	−0.10***									
**Level 2 (Class)**										
Sc positive motivation	−0.21					–	−0.22	0.97***	−0.88***	0.61***
Sc negative motivation	0.16						–	0.04	0.28	-0.24
Sc positive engagement	−0.34							–	−0.90***	0.70***
Sc negative engagement	0.12								–	−0.83***
Sc achievement	−0.26									–

*p < 0.05, **p < 0.05, ***p < 0.001.

#### Domain-General Motivation and Engagement

Domain-general academic motivation and engagement were assessed using the short form of the Motivation and Engagement Scale—High School (MES-HS; Martin, 2015). This measures all 11 factors represented in the Motivation and Engagement Scale, but does so via one item per factor. Single-item measures can present issues with reliability; however, because the larger research program from which these data emanate is focused on science, the full MES (44-items; see science motivation and engagement, above) was deemed too long. Therefore, the validated short form (Martin et al., 2015) was used. Also to note is research suggesting single-item scales have merit in cases where long scales are not able to be used (e.g., see Gogol et al., 2014).

Positive domain-general academic motivation constituted self-efficacy (“I believe I can do well in my schoolwork”), valuing (“What I learn in my schoolwork is important and useful”), and mastery orientation (“In my schoolwork, I am focused on learning and improving more than competing and being the best”). Positive engagement comprised planning behavior (“I plan out how I will do my schoolwork and study”), task management (“I use my study/homework time well and try to study and do homework under conditions that bring out my best”), and persistence (“I persist at schoolwork even when it is challenging or difficult”). Negative motivation comprised anxiety (“I get quite anxious about schoolwork and tests”), failure avoidance (“I mainly do my schoolwork to avoid failing or disapproval from parents or the teacher/s”), and uncertain control (“I don’t think I have much control over how well I do in my schoolwork”). Finally, negative engagement comprised self-handicapping (“In my schoolwork, I sometimes reduce my chances of doing well [e.g., waste time, disrupt others, procrastinate]”) and disengagement (“I often feel like giving up in my schoolwork”). Students rated items on a scale of 1 (Strongly Disagree) to 7 (Strongly Agree).

Because domain-general motivation and engagement refer to general academics (not just science or science lessons/classes), we modeled it at L1 (student-level), not at L2 (science class-level). As with science motivation and engagement, we focused on the 4 higher order MES factors (positive motivation, negative motivation, positive engagement, negative engagement) that were estimated by aggregating (mean scoring) the items of each higher order MES factor (e.g., the 3 items for positive motivation) to create 4 domain-general motivation and engagement scores and then deriving an error-adjusted mean score for each of these 4 scores. This was done using the same formula as was used for science motivation and engagement (see above; Hayduk, 1987; Cole and Preacher, 2014). This yielded standardized loadings as follows: positive domain-general motivation, L1 = 0.94; positive domain-general engagement, L1 = 0.94; negative domain-general motivation, L1 = 0.95; and negative domain-general engagement, L1 = 0.91. We found generally acceptable reliability for positive motivation (L1ω_h_ = 0.76), positive engagement (L1ω_h_ = 0.83), negative motivation (L1ω_h_ = 0.61), and negative engagement (L1ω_h_ = 0.57; to note is that this factor comprised only 2 items and fewer items attenuate reliability). Table 1 shows descriptive and reliability statistics for these factors.

#### Science Achievement

Science achievement was assessed using an online test. It comprised 12 questions developed by the science department head of a large Sydney school. Following preliminary item development, language accessibility was assessed by the languages department head (at the same school). To accommodate the different year-levels of participating students, two forms were developed, one based on the Stage 4 (years 7 and 8) state science syllabus and the other based on the Stage 5 (years 9 and 10) state science syllabus. Questions were set within the contexts of content strands Earth and Space, Physical World, Chemical World, and Living World (NSW Science Syllabus; Nsw Education Standards Authority, 2019). Thus, the questions aligned with students’ skill level and what they had been taught—and considered a snapshot of their scientific literacy. The two forms were considered by five experienced science teachers who reviewed each test item in terms of: (a) alignment with the state science syllabus, (b) language and cultural accessibility of item text/graphics, and (c) the envisaged percentage of students likely to correctly answer an item (response options: 25, 50, or 75% of students). All answers were recoded as 0 = incorrect and 1 = correct. The number of correct responses was summed to a total score (as a continuous scale), reflecting something of a formative construct, not a latent construct. Scores were standardized by year level (*M* = 0; *SD* = 1) so that students’ achievement scores were appropriately adjusted for different levels of science education and experience and for the fact two tests were administered (one test for years 7 and 8, raw *M* = 55 and 60%, respectively, one test for years 9 and 10, raw *M* = 52 and 57%, respectively). The science achievement factor was approximately normally distributed (Table 1), with acceptable reliability at L1 and L2 (L1ω_h_ = 0.69; L2ω_h_ = 0.98).

#### Background Attributes

Numerous background attributes were used as covariates and also as potential moderators of boarding effects. Participants reported their boarding status (0 = day student; 1 = boarding student), prior achievement (relative year-group standing on science tests and assignments; 1 = “in the lower third of my year group,” 2 = “in the middle third of my year group,” 3 = “in the upper third of my year group”), age (a continuous measure), gender (0 = male, 1 = female), language background (0 = English speaking, 1 = non-English speaking), Indigenous status (0 = non-Indigenous; 1 = Indigenous), parents’ education (scale from 1 “No formal qualifications” to 6 “university undergraduate or higher degree”), and SES based on home postcode which was then matched to Australian Bureau of Statistics SES values (a continuous score, ranging from relatively greater socio-economic disadvantage to relatively greater socio-economic advantage, national *M* = 1000, *SD* = 100). As described in section “Data Analysis” below, in the boarding sample we also examined the association between years as a boarding student (a continuous variable) and academic outcomes.

#### Personality

We were also interested in the extent to which boarding status accounted for variance beyond existing personality traits (in line with recommendations by Martin et al., 2014). A brief personality scale (Gosling et al., 2003) was administered, consisting in our study of a single item measure for each personality factor. Gosling et al. (2003) found adequate levels of validity and alignment between self and observer ratings. On a scale of 1 (Strongly Disagree) to 7 (Strongly Agree) students rated themselves on each of the “Big 5” personality traits, as follows: “I see myself as”… “sympathetic, warm” (agreeableness), “dependable, self-disciplined” (conscientiousness), “extraverted, enthusiastic” (extraversion), “anxious, easily upset” (neuroticism), “open to new experiences” (openness to experience).

### Data Analysis

The analyses were conducted with M*plus* 7.31 (Muthén and Muthén, 2015). Maximum likelihood with robustness to non-normality (MLR) was employed as the method of estimation (Muthén and Muthén, 2015). Missing data (7.75% missing data points) were dealt with via the M*plus* full information maximum likelihood defaults (FIML; Muthén and Muthén, 2015). To account for the fact that students (L1) and classrooms (L2) are clustered within schools, we also adjusted standard errors for school using the “cluster” and “complex” commands in M*plus* (we did not conduct a 3-level multilevel model—students nested within classrooms within schools—because there was not a sufficient number of schools to justify this).

In the first instance, variance components analyses were conducted to determine between-class variation in boarding status, and science motivation, engagement, and achievement. Intraclass correlations (ICCs) were of interest here, identifying the percentage of between-class variance for each measure (reported in Table 1). Then, multilevel correlation analyses were conducted, where, in a single model, student-level (L1) associations among all variables (domain-general and -specific) were examined, as were all relevant class-level (L2) associations (domain-specific only).

Following this, analyses centered on multilevel path analysis. This proceeded through three stages. For Step 1 at L1, student boarding status was entered as a predictor of student-level science motivation, engagement, and achievement and also student-level domain-general motivation and engagement. For Step 1 at L2, class-level boarding status predicted class-level science motivation, engagement, and achievement. For Step 2 at L1, student boarding status, background attributes, and personality factors were entered as predictors of student-level science motivation, engagement, and achievement and also student-level domain-general motivation and engagement. Step 2 at L2 was the same as Step 1 at L2. Step 3 at L1 added to Step 2 by also assessing the extent to which student-level background and personality attributes moderated the effects of student-level boarding status—by way of interaction terms (e.g., boarding × age, etc.; calculated by zero-centering the main effects and finding their product; Aiken et al., 1991). Step 3 at L2 (classroom-level) was the same as L2 in Steps 1 and 2. Boarding status was modeled using the doubly latent format in M*plus*, with L2 effects disentangled from L1 effects; however, for completeness, in Table 3 notes we present findings for a model in which boarding status was modeled as a raw score at L1 and a cluster (class) aggregate at L2—the same pattern of findings was derived. Figure 1 presents the complete model at Step 3.

**TABLE 3A:** Multilevel path model: Step 1 (boarding as predictor) and Step 2 (boarding, background attributes, and personality as predictors).

	Domain-general academic motivation and engagement				Science motivation and achievement				
	Positive motivation	Negative motivation	Positive engagement	Negative engagement	Positive motivation	Negative motivation	Positive engagement	Negative engagement	Achievement
**Step 1**									
**Level 1 (students)**									
Boarding	0.01	0.01	0.03	−0.02	−0.02	0.01	0.01	0.05**	−0.07***
**Level 2 (classrooms)**									
Boarding					−0.33	0.09	−0.29	0.24	−0.32*
**Step 2**									
**Level 1 (students)**									
Boarding	0.05**	−0.02	0.06***	−0.06	0.01	−0.02	0.02	0.03**	−0.04
Age	−0.09**	0.05*	−0.12***	0.19***	−0.16***	0.04	−0.16***	0.19***	−0.04
Gender (M/FM)	0.01	0.01	−0.05	0.05*	0.01	0.06*	0.01	0.03***	−0.04
Socio−econ status (SES)	0.04	−0.02	0.01	−0.02	−0.02	0.01	−0.03	0.04	−0.01
Non−English Speaking (NESB)	0.01	0.07***	0.03	−0.01	0.01	0.03	0.03	0.02	0.04
Indigenous	0.01	0.01	−0.01	0.04	0.01	−0.02	0.01	−0.02	−0.03*
Parent education	0.01	0.01	−0.01	0.01	0.03	0.02	−0.01	−0.01	0.08***
Prior achievement	0.10***	−0.14***	0.11***	−0.11***	0.17***	−0.20***	0.13***	−0.19***	0.37***
Neuroticism	−0.03*	0.50***	0.05**	0.31***	−0.01	0.38***	0.01	0.11***	−0.03
Openness	0.31***	−0.02	0.22***	−0.01	0.30***	−0.08***	0.22***	−0.19***	0.07
Conscientiousness	0.21***	0.01	0.34***	−0.19***	0.11***	−0.07	0.23***	−0.19***	0.04
Agreeableness	0.14***	−0.03	0.07*	0.05***	0.07**	−0.02	0.05**	−0.06*	0.03
Extraversion	0.05*	0.05	0.07*	−0.02	0.01	0.05	0.06**	0.04	−0.09**
**Level 2 (classrooms)**									
Boarding					−0.21	0.16	−0.34	0.12	−0.26
**Explained variance**									
Step 1 Level 1 *R*^2^	< 0.001	<0.001	< 0.01	<0.001	< 0.001	<0.001	< 0.001	<0.01	< 0.01
Step 1 Level 2 *R*^2^					0.11	< 0.01	0.08	0.06	0.10
Step 2 Level 1 *R*^2^	0.37***	0.30***	0.37***	0.22***	0.26***	0.25***	0.28***	0.25***	0.20***
Step 2 Level 2 *R*^2^					0.04	0.03	0.12	0.02	0.07

*p < 0.05, **p < 0.05, ***p < 0.001.

**TABLE 3B:** Multilevel path model: Step 3—Boarding, background attributes, personality, and interactions predicting motivation, engagement, and achievement.

	Domain−general academic motivation and engagement				Science motivation and achievement				
	Positive motivation	Negative motivation	Positive engagement	Negative engagement	Positive motivation	Negative motivation	Positive engagement	Negative engagement	Achievement
**Level 1 (students)**									
Boarding	0.05**	−0.01	0.08***	−0.09**	0.01	0.01	0.04	0.01	−0.04
Age	−0.09**	0.04*	−0.13***	0.18***	−0.16***	0.04	−0.16***	0.19***	−0.04
Gender (M/FM)	−0.01	0.01	−0.05	0.03*	0.01	0.06	0.02	0.04	−0.07
Socio−economic status (SES)	0.04	−0.03**	−0.02	−0.01	−0.04*	−0.02	−0.06***	0.07***	−0.06**
Non−English Speaking (NESB)	0.01	0.07***	0.02	−0.01	0.02	0.03	0.02	0.03*	0.04
Indigenous	0.01	0.02	−0.01	0.04	0.01	−0.02	0.01	−0.01	−0.02
Parent education	0.01	0.01	−0.01	0.01	0.03	0.02	0.01	−0.01	0.08***
Prior achievement	0.11***	−0.15***	0.11***	−0.10**	0.17***	−0.21***	0.12***	−0.19***	0.38***
Neuroticism	−0.04**	0.50***	0.05**	0.31***	−0.01	0.38***	−0.01	0.11***	−0.04
Openness	0.31***	−0.02	0.22***	−0.02	0.30***	−0.08***	0.22***	−0.19***	0.07
Conscientiousness	0.21***	0.01	0.34***	−0.19***	0.11***	−0.07	0.23***	−0.19***	0.04
Agreeableness	0.14***	−0.03	0.07*	0.05*	0.07*	−0.02	0.05*	−0.06	0.04
Extraversion	0.06**	0.05	0.08*	−0.02	0.01	0.05	0.07**	0.03	−0.08***
Board × Age	0.01	0.02	0.01	0.01	−0.01	−0.01	0.01	−0.01	−0.02
Board × Gender	−0.02	−0.01	−0.01	−0.03	0.01	0.01	0.03	0.01	−0.06***
Board × SES	0.01	0.02	0.04	−0.04	0.02	0.03	0.05	−0.07***	0.07*
Board × NESB	0.03	0.01	0.01	0.03**	0.03**	−0.02***	0.04***	0.02	0.01
Board × Indigenous	−0.01	−0.02	0.01	0.01	0.01	−0.01	−0.01	0.01	−0.04**
Board × Parent education	0.05	0.01	0.03	−0.01	0.03	−0.02	0.03	−0.04	0.01
Board × Prior achieve	−0.03*	0.03	−0.01	−0.01	−0.02	0.05	−0.01	0.04	−0.04**
Board × Neuroticism	0.02	0.02	0.01	0.01	0.02	0.01	0.01	0.01	−0.01
Board × Openness	−0.01	−0.04***	−0.03	0.02	−0.01	0.01	−0.02	−0.01	−0.02
Board × Conscientious	−0.01	0.06**	−0.01	0.10***	0.01	−0.01	0.02	0.02	−0.01
Board × Agree	0.02***	−0.01	0.01	−0.04	−0.02	−0.01	−0.03	0.04	0.05
Board × Extraversion	0.03**	0.02	0.03	−0.02	0.03	0.01	0.01	−0.02	−0.01
**Level 2 (Classrooms)**									
Boarding					−0.06	0.14	−0.14	0.10	−0.16
Explained variance									
Level 1 *R*^2^	0.38***	0.30***	0.37***	0.23***	0.26***	0.26***	0.28***	0.26***	0.21***
Level 2 *R*^2^					< 0.01	0.02	0.02	0.01	0.03

*p < 0.05, **p < 0.05, ***p < 0.001. Our study employed a doubly *latent* approach to model boarding at L1 and L2, but for completeness we ran a Step 3 model where boarding at L1 was a raw score and boarding at L2 was a cluster (class) aggregate of this L1 raw score. The same pattern of L1 and L2 boarding effects were found: L1 boarding status → domain-general positive motivation, β = 0.05, p < 0.01; L1 boarding status → domain-general negative motivation, β = −*0.02, p = 0.626; L1 boarding status → domain-general positive engagement, β = 0.08, p < 0.001; L1 boarding status → domain-general negative engagement, β = −0.10, p < 0.01; L1 boarding status → science positive motivation, β = 0.01, p = 0.811; L1 boarding status → science negative motivation, β = 0.01, p = 0.471; L1 boarding status → science positive engagement, β = 0.03, p = 0.429; L1 boarding status → science negative engagement, β = 0.02, p = 0.528; L1 boarding status → science achievement, β* = −*0.03, p = 0.193; L2 boarding status → science positive motivation, β* = −*0.12, p = 0.141; L2 boarding status → science negative motivation, β* = −*0.06, p = 0.832; L2 boarding status → science positive engagement, β* = −*0.06, p = 0.638; L2 boarding status → science negative engagement, β* = −*0.01, p = 0.949; L2 boarding status → science achievement, β* = −*0.20, p = 0.122.*

In a supplementary analysis among boarding students only, we also investigated the association between years in boarding school and academic outcomes. At L1, years in boarding (alongside background attributes, and personality factors) was entered as a predictor of student-level science motivation, engagement, and achievement and also student-level domain-general motivation and engagement. At L2, class-level years boarding (i.e., average years boarding in a class) predicted class-level science motivation, engagement, and achievement.

In our study, the sample is large and there is a risk that effects are disproportionately biased toward statistical significance. Thus, to avoid giving undue weight to effect sizes that are statistically significant but small (given the large sample size), we applied Keith’s (2006) guidelines and a more stringent *p*-value (*p* < 0.001) to help us determine if a finding was interpretable. As per Keith (2006), effect sizes (β) of 0.05 and above are considered small, β of 0.10 and above are moderate, and β of 0.25 and above are large. Accordingly, effects that are significant at *p* < 0.001 and β ≥ 0.05 are taken as interpretable.

## Results

### Descriptive Statistics, Classroom Variation, and Multilevel Correlations

Table 1 presents means, standard deviations, skewness, kurtosis, and reliability (coefficient omega) for all substantive measures (motivation, engagement, achievement) in the study. Socio-demographic descriptive statistics were presented in Participants, above. The distributional properties demonstrated that the measures were approximately normally distributed, with standard deviations appropriately proportional to means, and skewness and kurtosis values within acceptable ranges (Kline, 2011). Omega coefficients ranged between 0.61 and 0.84 at student-level (L1) and between 0.87 and 0.98 at classroom-level (L2), suggesting generally acceptable reliability (McNeish, 2018).

Variance components analyses identified the between-class variation (i.e., difference between science classrooms) in boarding status, science motivation, science engagement, and science achievement. Findings are shown in Table 1 which presents intraclass correlations (ICCs) and indicate the percentage variance for these variables from class-to-class (i.e., the percentage of how much variation there is between science classrooms, relative to residual and student-to-student variation). ICCs for the study’s L2 variables were: boarding status = 0.15 (15%), positive science motivation = 0.14 (14%), positive science engagement = 0.09 (9%), negative science motivation = 0.08 (8%), negative science engagement = 0.14 (14%), and science achievement = 0.31 (31%). There is thus notable variation between classrooms on each of the L2 factors—and with more than 10% of the variance on most factors explained at Level 2, multilevel modeling was justified (Byrne, 2012).

We proceeded to test multilevel correlations underlying the hypothesized multilevel path model. This generates bivariate correlations that are the first insight into the relationships tested in Figure 1. Correlations are presented in Table 2. Here we summarize only significant correlations with L1 and L2 boarding factors (all non-significant correlations and all correlations among background attributes, personality, and outcomes are in Table 2). For L1 we examine the association between students’ boarding status and their motivation, engagement, and achievement; with positive (or negative) correlations indicating boarders scoring higher (or lower) on motivation, engagement, and/or achievement. For L2 we examine the association between the proportion of boarding students in a classroom and class-average motivation, engagement, and achievement; with positive (or negative) correlations indicating classrooms with a higher (or lower) proportion of boarders scoring higher (or lower) on class-average motivation, engagement, and/or achievement. As noted in Data Analysis, given the number of participants and the many parameters tested, we here focus on effects attaining *p* < 0.001. At the student-level (L1), boarding status was significantly and positively correlated with age (*r* = 0.14, *p* < 0.001; boarders older), SES (*r* = −0.59, *p* < 0.001; boarders lower), parent education (*r* = −0.12, *p* < 0.001; boarders lower), prior achievement (*r* = −0.14, *p* < 0.001; boarders lower), agreeableness (*r* = −0.05, *p* < 0.001; boarders lower), and science achievement (*r* = −0.10, *p* < 0.001; boarders lower). At L2 (classroom-level), boarding status was not significantly correlated with any outcome factors.

### Multilevel Path Analyses

#### Step 1 Main Effects

In Step 1 at student-level (L1), boarding status was the sole predictor of domain-general motivation and engagement and science motivation, engagement, and achievement. At classroom-level (L2), boarding status (proportion of boarders in a classroom) was the predictor of class-average science motivation, engagement, and achievement. In all cases, positive (or negative) standardized beta values indicate that boarding is associated with higher (or lower) scores on academic outcomes. Multilevel path analysis showed that student-level (L1) boarding status predicted science achievement (β = −0.07, *p* < 0.001; boarders lower) and negative science engagement (β = 0.05, *p* < 0.01; boarders higher). However, only the effect for science achievement attained the dual criteria for interpretability [β ≥ 0.05 (as per Keith, 2006) and *p* < 0.001—see section “Data Analysis” above]. For Step 1 at the class-level (L2), boarding status did not significantly predict any L2 science motivation, engagement, or achievement factors. Thus, the number of boarding students in the class (relative to day students) was not differentially associated with academic motivation, engagement, and achievement. In keeping with these generally non-significant boarding effects, the variance explained (*R*^2^) in Step 1 is also low. All Step 1 findings (significant and non-significant) are presented in Table 3A.

#### Step 2 Main Effects

In Step 2 at student-level (L1), boarding status, background attributes, and personality were predictors of domain-general motivation and engagement and science motivation, engagement, and achievement. At classroom-level (L2), boarding status (proportion of boarders in a classroom) was the predictor of class-average science motivation, engagement, and achievement. In all cases, positive (or negative) standardized beta values indicate that boarding is associated with higher (or lower) scores on academic outcomes. All (significant and non-significant) findings are presented in Table 3A. Here we focus on boarding effects; effects for all other predictors are shown in Table 3A. These analyses showed that student-level (L1) boarding status predicted positive domain-general motivation (β = 0.05, *p* < 0.01; boarders higher), positive domain-general engagement (β = 0.06, *p* < 0.001; boarders higher), and negative science engagement (β = 0.03, *p* < 0.01; boarders higher). However, only the effect for positive domain-general engagement attained the dual criteria for interpretability; and, the interpretable Step 1 effect for achievement dropped out. Class-level (L2) boarding status did not significantly predict any L2 science motivation, engagement, or achievement factors. Thus, the proportion of boarding students in the class was not significantly associated with class-average academic motivation, engagement, and achievement.

Inclusion of Step 2 background and personality attributes yielded a significant increase (at *p* < 0.001) in explained variance for L1. Thus, at L1 for domain-general outcomes, beyond the role of boarding status these student attributes explained significant variance in positive motivation (*R*^2^ = 0.37), negative motivation (*R*^2^ = 0.30), positive engagement (*R*^2^ = 0.37), and negative engagement (*R*^2^ = 0.22). At L1 for science outcomes, beyond the role of boarding status the student attributes explained significant variance in positive motivation (*R*^2^ = 0.26), negative motivation (*R*^2^ = 0.25), positive engagement (*R*^2^ = 0.28), negative engagement (*R*^2^ = 0.25), and achievement (*R*^2^ = 0.20).

#### Step 3 Main and Interaction Effects

In Step 3 at student-level (L1), boarding status, background attributes, personality (as main effects) and the cross-products of boarding × background/personality attributes (interaction effects; e.g., boarding × age, etc.) were predictors of domain-general motivation and engagement and science motivation, engagement, and achievement. At classroom-level (L2), boarding status (proportion of boarders in a classroom) was the predictor of class-average science motivation, engagement, and achievement. In all main effects, positive (or negative) standardized beta values indicate that boarding is associated with higher (or lower) scores on academic outcomes. Interaction effects are unpacked as appropriate and described below. All (significant and non-significant) findings are presented in Table 3B.

For Step 3 *main effects*, multilevel path analysis showed that student-level (L1) boarding status predicted positive domain-general motivation (β = 0.05, *p* < 0.01; boarders higher), domain-general positive engagement (β = 0.08, *p* < 0.001; boarders higher), and negative domain-general engagement (β = −0.09, *p* < 0.01; boarders lower). However, only the effect for positive domain-general engagement attained the dual criteria for interpretability. Class-level (L2) boarding status did not significantly predict any L2 science motivation, engagement, or achievement factors. In this final model, other L1 main effects attaining the dual criteria for interpretability were age, prior achievement, neuroticism, openness, conscientiousness, and agreeableness (see Table 3B for strength and direction of standardized beta coefficients).

For Step 3 *interaction effects*, three effects attained Keith’s (2006) benchmark (β ≥ 0.05) and significance at *p* < 0.001. The first was boarding × conscientiousness for negative domain-general engagement (β = 0.10, *p* < 0.001). In follow-up simple effects tests, we found that for students low in conscientiousness there was a larger effect of boarding status on negative domain-general engagement (β = −0.09) than for students high in conscientiousness (β = −0.01). The second was boarding × gender for science achievement (β = −0.06, *p* < 0.001). For females, there was a larger effect of boarding status on science achievement (β = −0.18) than for boys (β = −0.04). The third was boarding × SES for negative science engagement (β = -0.07, *p* < 0.001). For low SES students, there was a larger effect of boarding status on negative science engagement (β = 0.04) than for high SES students (β < 0.01).

#### Supplementary Analyses: Years as a Boarding Student

In a supplementary analysis among boarding students only, we also investigated the association between years as a boarding student and academic outcomes. Controlling for background attributes and personality factors at L1, we found that years as a boarding student positively predicted science test achievement (β = 0.08, *p* < 0.01; more time in boarding associated with higher achievement); however, this effect did not attain our dual criteria for interpretability (β ≥ 0.05 and *p* < 0.001). Class-level (L2) years in boarding did not significantly predict any L2 science motivation, engagement, or achievement factors. Taken together, then, time spent in boarding was not a salient factor in students’ academic outcomes.

## Discussion

After controlling for background and personality attributes, we found predominant parity between boarding and day students in their motivation, engagement, and achievement. We also found that motivation, engagement, and achievement at the class-level were not significantly affected by the number of boarders in the classroom. In addition, the effects of boarding were generally not moderated by students’ background or personality attributes. Thus, we conclude that boarders have academic opportunities and outcomes that are comparable to day students.

### Student-Level and Classroom-Level Effects: Boarding vs. Day Status

Schools comprising boarding and day students represent a unique research design. In our study, many students constituted what we might consider a “treatment” group (boarding students) and many others constituted a “comparison” group (day students). The two groups were educated in the same classrooms and received the same syllabus and instruction from the same teachers. In fact, the clustering of students in the same classrooms enabled us to extend prior research by investigating the extent to which the number of boarders in a (science) classroom had an impact on class-average motivation, engagement, and achievement outcomes (in science).

Prior multivariate research into boarding effects had only considered domain-general motivation and engagement (Martin et al., 2014), and found predominant parity between boarding and day students on these outcome factors. However, that earlier research had been conducted at the individual student level, looking at an individual’s boarding (or day) status and its relationship with an individual’s motivation and engagement; it did not take into account the possibility that a critical mass of boarding students in a classroom may affect class-level outcomes. It is known that individuals can affect the group to which they belong and in turn the group can affect these individuals (Raudenbush and Bryk, 2002; Goldstein, 2003; Marsh et al., 2008). This raises the question: given that boarding status represents a unique educational experience (see section “Introduction”), does that experience converge in a classroom where other boarders are present to affect overall class-average outcomes? Because we collected data on science motivation, engagement, and achievement in science classrooms, we were able answer this question. Our findings showed that the proportion of boarders in the classroom (relative to day students) was not significantly associated with class-average science outcomes.

The study’s multilevel design was important to help better ascertain the nature of boarding effects. With this design we could disentangle student-level (Level 1) variance from class-level (Level 2) variance. In doing so it was evident that boarding status was not associated with science outcomes at either level (Table 3B). Given this, it was interesting to note that there were boarding effects for domain-general motivation and engagement (boarders higher in positive domain-general motivation and engagement and lower in domain-general negative engagement)—though only one (for domain-general positive engagement) attained our dual criteria of interpretability (β ≥ 0.05 and *p* < 0.001). In our study, positive engagement comprised task management, planning and monitoring, and persistence factors. These are behavioral dimensions that may be quite responsive to the structured nature of study conditions in boarding contexts (Lee and Barth, 2009). In these contexts, there are typically well-organized and well-planned study times and routines that students work to. These activities are also overseen and supported by teachers or other boarding house staff. Over time and relative to day students, these factors may have the effect of promoting a general disposition to better task management, persistence, etc. (i.e., positive domain-general engagement). Moreover, over time, boarding students may come to internalize these behaviors as their own capacities, further contributing to higher self-reported domain-general positive engagement. Interestingly, this was not the case for the domain-specific counterpart (positive science engagement) and this may be because there are key aspects of science engagement that are class-specific and applicable to both day and boarding students (e.g., science practicums, experiments, predicting, observing, etc.) and not linked to boarding study regimes.

### Background and Personality Attributes

Findings in this study were also notable because they represented effects after controlling for background and personality attributes. As shown in correlations in Table 2, there were significant bivariate associations among background attributes, boarding status, and academic outcomes—suggesting a need in our multivariate modeling to account for variance attributable to background attributes when assessing the unique relationship between boarding status and academic outcomes. In fact, Martin et al. (2014) emphasized the need for this and so our findings continue to underscore the fact that boarding effects cannot be fully interpreted without considering students’ background and personality attributes. Future research investigating boarding effects might thus consider these as particularly important to include and control for. Taken together, findings suggest that researchers ought not confuse or conflate boarding effects with effects due to some key background and personality attributes of boarding students. Relatedly, researchers ought to avoid raw comparisons of boarding and day students. Without adjusting for relevant background and personality attributes, raw comparisons may lead to biased results.

It has also been suggested that there may be some students for whom boarding may offer particular benefit. For example, it may provide Indigenous, rural, or remote students with educational opportunities not available to them in their distant residential communities. Or, being an inherently social residential context, perhaps boarding is better suited to students high in extraversion. We were able to test these possibilities through interaction effects (e.g., boarding × Indigenous status; boarding × extraversion, etc.; Table 3). Our findings suggested that for the most part boarding effects were not moderated by students’ background and personality attributes. Of the 108 possible interaction effects, only three attained our benchmark for interpretability (β ≥ 0.05 and *p* < 0.001): boarding × conscientiousness, boarding × gender, and boarding × SES. Taking our main and interaction effects together, then, it appears that including background and personality factors as main covariate effects is important when understanding boarding status, but it may not be necessary to model these background and personality factors as moderators of boarding status. Future research may seek to confirm this.

### Practice Implications

We suggest that in the context of commentary and research documenting adversity for students in boarding schools, our finding of educational parity between boarding and day students is notable and has implications for educators and parents. For parents, one of their main concerns is that their child has educational opportunities and access on par with other students in a school. Indeed Lawrence (2005) identified that parents choose to board their child for various opportunities (e.g., extracurricular activity) and a structured and stable learning environment. Our findings suggest they receive such support—at least, to the extent that they evince academic outcomes comparable with day students. Many parents also send their child to board because, for one reason or another, their child does not have optimal educational access (e.g., due to geographic distance, etc.). We found that boarders’ results on motivation, engagement, and achievement were comparable to that of day students and we infer that this reflects equal opportunity and access for boarding students.

It was also interesting to note that the proportion of boarders in a classroom did not seem to be associated with class-average motivation, engagement, and achievement. There were no significant differences in these academic outcomes as a function of whether there were fewer or more boarders in the class. Schools often wrestle with classroom composition and how to collect students together to optimize academic and other outcomes. There has been a small body of research investigating classroom composition, finding some evidence that there are differences in motivation and engagement between classes taught by the same teacher (Marsh et al., 2008). Our study adds to this work and would suggest that schools need not factor in the ratio of boarders to day students when deciding on class composition.

On the issue of access and opportunity, the general lack of moderation effects suggested no differences in academic outcomes between subgroups of boarders (and day students). For example, as we explained in the introduction, there have been questions about whether cultural identity may be unduly affected by the boarding experience or whether gender may play out in problematic ways in boarding contexts. In our study, there appeared to be no problematic patterns of interaction effects that would suggest issues along these lines: the general parity in academic outcomes between boarding and day students was found irrespective of a student’s background and personality attributes. From a practice perspective, the general lack of moderation effects suggests that efforts aimed at promoting motivation, engagement, and achievement among boarders need not be differentially directed at different sub-groups within the boarding community. Put another way, whether a student is a boarder or not, educational support to compensate low SES status or low prior achievement is required. Nevertheless, we did not assess some other potentially influential background attributes such as learning difficulties, which may require particular attention for some boarders (but conceivably not any more or less than among day students with learning difficulties, which would again suggest no interaction effect).

Importantly, however, although our study found predominant parity between boarders and day students and no interaction effects of note, there is no question that the transition from one’s community (and day school) to boarding school is a major one (Martin et al., 2014). This being the case, it is prudent to consider educational support that can assist boarders in this transition and then through school. It is noteworthy that recent research in the Australian context (with particular focus on Indigenous students) has conducted quite a substantial body of work identifying supports that may be helpful. For example, research has shown that multidimensional intervention can be effectively administered to promote the resilience of Indigenous boarding students (Benveniste et al., 2020). Likewise, a study of a social-emotional wellbeing program found that Indigenous boarders experienced an enhanced capacity to seek and provide help, work in groups, manage conflict, and discuss cultural issues (Franck et al., 2020; see also Heyeres et al., 2018; Rutherford et al., 2019). Practices within the boarding school can also provide further opportunities to assist boarders’ academic and social-emotional wellbeing. For example, it has been shown that boarding staff can harness positive relationships with students to enhance students’ educational participation, mental health, and self-responsibility (McCalman et al., 2020). Qualitative data from Indigenous boarding students and staff have also identified how boarding schools can be physically designed to optimize a sense of belonging. These include flexible spaces to foster relationships inside the boarding house, student voice in how spaces are designed and arranged, and spaces that provide “cultural relief” (Whettingsteel et al., 2020). There are also culturally based strategies that can support boarding outcomes. For example, Lloyd and Duggie Pwerl (2020) showed how Indigenous students can achieve Western educational success in a boarding context through efforts by the school to maintain key aspects of their culture (also see Bobongie, 2017). Relatedly, Osborne et al. (2019) discuss this idea in terms of “both ways” capital where educators seek to affirm and strengthen Indigenous identity *and* help them develop positive Western academic codes.

### Limitations, Future Directions, and Conclusions

There are limitations in our study important to consider when interpreting findings and which offer some direction for future research. First, we speculated that the boarding context comprises routines, structures, and interactions with educational support staff that are unique to that context—and that this may yield particular educational effects relative to day students. However, we did not have data on these factors in this study. We also did not have data on where boarders were from, including cohorts within the boarding sub-sample (such as Indigenous students). We therefore do not know if boarders from different areas evince different motivation, engagement, and achievement patterns and we cannot as fully contextualize the findings derived in this study. In future, collecting such data and ascertaining their impacts will identify more distinct effects particular to the boarding context and to whom these effects apply. Second, our study focused on high school students, not younger students in elementary school settings. Theories of attachment (e.g., Ainsworth and Bowlby, 1991) have emphasized the influential role of parents in children’s lives and it is possible that boarding reduces these important influences and may stunt personal development for younger students (also see Jack, 2020). It is thus important to test the generalizability of our findings to students in a range of other educational and residential contexts.

Third, given our cross-sectional data, there are questions about factor and causal ordering which may be answered by collecting data that monitors students who move from day status to boarding status and vice versa, as well as collecting data over time (i.e., longitudinal). This would provide a unique perspective on what changes (if any) occur as a result of changing status from one to the other. Relatedly, future research might look to include social-emotional wellbeing indicators as background attributes to disentangle the role of boarding status on academic outcomes from prior social-emotional wellbeing. These could include measures such as the Flourishing Scale (Diener et al., 2009) and Kessler Psychological Distress Scale (Kessler and Mroczek, 1994). Although our study did control for trait-like personality (including neuroticism as a mental health indicator), it is important to also control for more specific social-emotional wellbeing state-like measures. Indeed, the importance of considering a diversity of key factors and issues of modeling and causal ordering are increasingly being recognized by boarding school researchers (e.g., Guenther et al., 2020). Fourth, there is a need for more intensive real-time data. Recent research using mobile technology to capture real-time motivation and engagement has revealed in-the-moment variance in motivation and engagement (Martin et al., 2015, 2019b) and it would be fascinating to explore motivation and engagement while students are doing study and homework in the boarding house. It is also important to recognize measurement issues for some groups of students in the boarding sector. For example, Langham et al. (2018) identified some challenges validating previously established tools measuring resilience among Indigenous boarders. It is therefore encouraging that the motivation and engagement measurement tool used in this study has been validated among Indigenous students (Martin et al., 2019a) and has previously been effective in assessing boarding effects among Indigenous students (Martin et al., 2014). Fifth, despite modernization of the boarding sector (Anderson, 2005), there are students for whom it is a negative experience. Future research might conduct person-centered analyses (e.g., latent profile analysis) to explore potential subgroups of boarders for whom it is a negative experience and examine the reasons why and the impact of this negative experience on their academic outcomes.

Sixth, as noted in Methods, the majority of students in this study were boys. Because of this, we conducted numerous additional statistical analyses leading us to tentatively conclude that gender composition did not disproportionately or unreasonably impact factors and empirical associations in this study. It is also the case that the sample was generally higher in SES than the national average; also, there were markedly more day students than boarding students. In some ways these imbalances are unavoidable in the Australian context as boarding schools tend to be higher SES independent schools and the ratio of boarding-to-day students is somewhat disproportionate given that most Australian boarding schools are in major urban areas or regional centers and enroll many “local” day students. We also point out that the average level of SES for our boarders was around the national average (see section “Methods”). Nevertheless, our investigation of interaction effects was important here because it allowed us to ascertain boarding and day status effects as a function of low and high SES. Another important feature of analyses was our modeling of SES as a covariate in analyses to control for variation attributable to it when seeking to identify unique boarding effects (beyond, for example, their lower SES relative to day students). This yielded a finding of predominant parity between boarding and day students. Thus, in the context of a history of negative effects of boarding on young people’s development (Duffell, 2000), our finding of predominant parity is significant. This notwithstanding, future research should recruit more balanced samples to be further assured of the generalizability of the present results, as well as to look at potential gender differences. Finally, we did not have enough schools to model at the school-level; we could only do so at the student-level (for domain-general and domain-specific outcomes) and class-level (for domain-specific outcomes). We note prior research found variation between schools in their capacity to support boarders (McCalman et al., 2020). Future research might recruit a sufficient number of schools to explore any variation in outcomes at the school-level. In all these ways we can better understand and assist boarding students as they navigate through their residential educational experience.

## Data Availability Statement

The datasets presented in this article are not readily available because: Part of an industry research partnership; consent from participants to share dataset not available; summative data (e.g., correlation matrix with standard deviations) available to enable analyses. Requests to access the datasets should be directed to AM, andrew.martin@unsw.edu.au.

## Ethics Statement

The studies involving human participants were reviewed and approved by the UNSW Human Ethics Committee. Written informed consent to participate in this study was provided by the participants’ legal guardian/next of kin.

## Author Contributions

AM led research design, led data analysis, and led report writing. EB and RK assisted with research design, assisted with interpretation of findings, and assisted with report writing. JP assisted with research design and assisted with report writing. VM-S assisted with report writing. All authors contributed to the article and approved the submitted version.

## Conflict of Interest

It is appropriate to note that one of the measures (the MES) in the study is a published instrument developed by this study’s first author attracting a small fee and royalty, part of which is put toward its ongoing development and administration and part of which is also donated to UNICEF. However, for this study, there was no fee involved for its use. The authors declare that the research was conducted in the absence of any commercial or financial relationships that could be construed as a potential conflict of interest.

## Footnotes

1 Boarding students are situated in residential contexts, often called boarding houses, boarding dormitories, or boarding residences (referred to henceforth as boarding houses). Boarding schools comprise one or several boarding houses. The ratio of boarding-to-day students in a school varies. Some schools are mainly day schools, with relatively few boarding students; other schools have a larger boarding contingent.
